# Data on spot–kits versus titration method for iodine determination in salt: Performance and validity

**DOI:** 10.1016/j.dib.2018.10.001

**Published:** 2018-10-04

**Authors:** Hamid Reza Shamsollahi, Noushin Rastkari, Maryam Nadarloo, Sara Sadat Hosseini, Razieh sheikhi, Ramin Nabizadeh

**Affiliations:** a Department of Environmental Health Engineering, School of Public Health, Tehran University of Medical Sciences, Tehran, Iran; b Center for Air Pollution Research (CAPR), Institute for Environmental Research (IER), Tehran University of Medical Sciences, Tehran, Iran

**Keywords:** Iodine spot-kit, Iodine deficiency disorder, Salt iodization

## Abstract

The aim of this data is comparison of achieved data from salt iodine measurement by titration method with using sodium thiosulfate in presence of lugol׳s reagent and commercial spot- kit. Titration measurement was carried out in two different laboratories using standard samples. 437 samples including 20 commercial brands were collected throughout Iran. The iodine contents of the samples were measured by both the titration method and two most frequently used spot-kit brands in Iran. There is no significant differences between the results obtained from the two brands of spot-kits (ICC = 0.797). The kits sensitivity for determination of negative samples was high (more than 0.9) but by increasing the iodine concentration up to 15 ppm, the kits’ sensitivity was decreased. These findings indicate that the titration method is necessary for quantitative purposes, especially for concentrations higher than 30 ppm. However, spot-kits are suitable for qualitative and semi-quantitative measurements.

**Specifications table**

**Table:** 

Subject area	Food Chemistry
More specific subject area	Iodine determination
Type of data	Tables, Figure
How data was acquired	20 random commercial brands including 437 samples were gathered around the Iran and their iodine contents were measured by titration method in two different labs for validation of measured values.
Data format	Raw, Analyzed
Experimental factors	The mentioned parameters above, in abstract section, were analyzed according to the standards.
Experimental features	Iodine determination in salt
Data source location	Tehran University of Medical Sciences, Tehran, Iran
Data accessibility	The data are available with this article
Related research article	Pandav CS, Arora NK, Krishnan A, Sankar R, Pandav S, Karmarkar MG. Validation of spot-testing kits to determine iodine content in salt. Bulletin of the World Health Organization. 2000;78(8):975-80.

**Value of the data**

• This data can be used for insure health managers that spot kits have appropriate validity for salt iodine concentration monitoring programs.
• As this data is shown, spot-kits are cost effective method for rapid monitoring of salt iodine concentration in low income countries.
• This data is useful for comparison of available commercial spot-kits, produced around the world for determination of invalid commercial brands.

## 1 Data

The values of iodine content of real samples measured by both the reference method and the spot-kits A and B are shown in Table 1, Table 2, in four different ranges: 0 to ≤8, 8 to ≤15 ,15 to ≤30 and >30 ppm. Linear regression fit of the iodine values measured by the titration method, as reference method, in two different labs gave an R^2^ value of 0.9537. Therefore results achieved from the titration method are valid. Table 3, Table 4 contain sensitivity, specificity, positive predictive values (PPV) and negative predictive values (NPV) of the spot-kit A and spot-kit B. Table 3, Table 4 have two categories including presence or absence of iodine (0 ppm or not) and presence or absence of significant amount of iodine (≤ 15 ppm or > 15 ppm). In order to compare the two brands of spot-kits, the interclass correlation (ICC) analysis was performed using the results shown in Table 1, Table 2. The outcome of ICC is summarized in Table 5.

**Table 1:** Comparison of the titration method and spot-kit A in determination of iodine content in salt.

Spot-testing kit A (ppm of iodine)	Iodine concentration by iodometric titration (ppm)				Total
	0 to ≤ 8	8 to ≤ 15	15 to ≤ 30	≥ 30	
0 to ≤ 8	19	10	55	41	125(28)
8 to ≤ 15	2	6	88	101	197(45)
15 to ≤ 30	0	0	37	78	115(26)
≥ 30	0	0	0	0	0(0)
Total	21 (4.8)	16(3.66)	180(41.1)	220(50.34)	437

**Table 2:** Comparison of the titration method and spot-kit B in determination of iodine content in salt.

Spot-testing kit B (ppm of iodine)	Iodine concentration by iodometric titration (ppm)				Total
	0 to ≤ 8	8 to ≤ 15	15 to ≤ 30	≥ 30	
0 to ≤ 8	19	9	56	38	122(27)
8 to ≤ 15	2	7	85	100	194(44)
15 to ≤ 30	0	0	39	82	121(27)
≥ 30	0	0	0	0	0(0)
Total	21(4.8)	16(3.66)	180(41.1)	220(50.34)	437

**Table 3:** Spot-kit A validation using as a qualitative method.

Interpretation of test	Indicators			
	Sensitivity	Specificity	PPV^a^	NPV^b^
Iodine absence (0 ppm)	0.96 (0.93–0.97)	0.78 (0.4–0.97)	0.99 (0.98–1)	0.06 (0.03–0.1)
Iodine presence (>0 ppm)				
Iodine deficiency(≤ 15 ppm)	0.76 (0.72–0.8)	0.85 (0.68–0.95)	0.98 (0.96–0.99)	0.23 (0.15–0.31)
Adequate iodine (> 15 ppm)				

a Positive predictive value.

b Negative predictive value.

**Table 4:** Spot-kit B validation using a qualitative method.

Interpretation of test	Indicators			
	Sensitivity	Specificity	PPV	NPV
Iodine absence (0 ppm)	0.91 (0.88–0.94)	0.89 (0.52–1)	1 (0.99–1)	0.19 (0.08–0.31)
Iodine presence (>0 ppm)				
Iodine deficiency(≤ 15 ppm)	0.76 (0.72–0.81)	0.82 (0.65–0.93)	0.98 (0.96–0.99)	0.23 (0.15–0.31)
Adequate iodine (> 15 ppm)

**Table 5:** Summary of the ICC analysis of the results obtained from the two spot-kits.

**Kinds of test**	**Agreement**
Test model	One way
Number of samples	437
ICC	0.797
Rater	2
Confidence interval	0.95
ICC range	0.761–0.829
α	0.887

## 2 Experimental design, material and methods

### 2.1 Setting up a calibration plot using the reference method

In order to set up a calibration plot using the titration method, as reference method, 200 g NaCl (analytical grade, MERK, GERMANY) was dissolved in 1000 mL distillated water and divided into four 250 mL aliquots. 10, 20, 40 and 80 µg potassium iodate (KIO_3_) were added to each of the aliquots. Then, 1 mL H_2_SO_4_ (2 N) and 5 mL KI (10% w/v) were added to each aliquot and the mellow yellow color appeared. After 10 minutes in the dark, the solutions were titrated by 0.005 N Na_2_S_2_O_3_ until light yellow color appeared. Then some drops of lugol׳s iodine indicator were added and titration was continued until the solution turned colorless. The consumed volume of Na_2_S_2_O_3_was used to calculate iodine concentration according to Eq. (1) [1], [2], [3], [4], [5], [6], [7], [8], [9], [10]:

(1) $$I=(\frac{V\times F\times 0.1058}{W})\times 1000$$

*I* = iodine concentration (µg/kg), *V* = consumed volume of Na2S2O3 (mL), *F* = modifying coefficient for Na_2_S_2_O_3_ solution, *W* = weight of dry salt sample (g)

The measured values were fitted against the real values of standard solutions in a linear regression model and the *R*^2^ was achieved.

### 2.2 Measurement of the iodine contents of real samples

After setting up a calibration plot using the titration method, iodine contents of real samples were measured by titration method. In this step, 20 random commercial brands including 437 samples were gathered around the Iran and their iodine contents were measured by this method in two different labs for validation of measured values.The results obtained in both labs were fitted in a linear regression model. Achieved *R*^2^ was 0.993 that shows there is not any significant differences between achieved data in two labs.

In the next step, the iodine content of all the samples were measured by two available brands of spot-kits. To this purpose, one drop of each kit׳s indicator was added to the samples to produce color. Produced color was compared to a color standard series. In order to determine the degree of agreement between two kits, the interclass correlation (ICC) statistical analysis was done. Also, sensitivity, specificity, positive predictive value (PPV) and negative predictive value (NPV) were calculated for determination of validity of results obtained from the kits. All the statistical analyses were done in R software environment (version 3.4.3).
